# An observational study ascertaining the prevalence of bullae and blebs in young, healthy adults and its possible implications for scuba diving

**DOI:** 10.3389/fphys.2024.1349229

**Published:** 2024-02-14

**Authors:** Max F. Bresser, Thijs T. Wingelaar, Jaap A. F. Van Weering, Paul Bresser, Rob A. Van Hulst

**Affiliations:** ^1^ Department of Respiratory Medicine, OLVG, Amsterdam, Netherlands; ^2^ Department of Anesthesiology, Amsterdam University Medical Center, Amsterdam, Netherlands; ^3^ Diving and Submarine Medical Center, Royal Netherlands Navy, Den Helder, Netherlands; ^4^ Department of Radiology, OLVG, Amsterdam, Netherlands

**Keywords:** air-filled cavities, intrapulmonary cavities, fitness to dive, computed tomography-CT, epidemiology

## Abstract

**Introduction:** Intrapulmonary air-filled cavities, e.g., bullae, blebs, and cysts, are believed to contribute topulmonary barotrauma (PBT) and arterial gas embolism (AGE) in divers. However, literature is unclear about the prevalence of bullae in healthy adults, ranging from 2.3—33.8%. While this could in part be explained due to increasing quality of radiologic imaging, such as computed tomography (CT) scans, other methodological factors may also affect these findings. This study aims to ascertain the prevalence of bullae in young and healthy adults.

**Methods:** This single-center cross-sectional observational study re-assessed the CT scans of adults (aged 18—40) performed for a clinical suspicion for pulmonary embolism, from 1 January 2016 to 1 March 2020. Presence of bullae was recorded in an electronic database. Chi-square and Fisher exact tests were used for statistical analyses. Additionally, a multivariate logistic regression analysis was performed to study the independent predictive value of identified risk factors.

**Results:** A total of 1,014 cases were identified, of which 836 could be included. Distribution amongst age groups (18–25, 26–30, 31–35, and 36–40) was almost equally, however, 75% of the population was female. Of the male proportion, 41% smoked, compared to 27% in females. In 7.2% (95% CI 5.6–9.1) bullae were identified. The prevalence increased with increasing age (*p* < 0.001), with odd ratios up to 5.347 (95% CI 2.164–13.213, *p* < 0.001) in the oldest age group. Males and smokers had higher odds ratios for bullae of 2.460 (95% CI 1.144–4.208; *p* = 0.001) and 3.406 (95% CI 1.878–6.157, *p* < 0.001), respectively. Similar results were seen in the multivariate logistic regression analysis, where age, male sex and smoking were all statistically significant independent risk factors for bullae.

**Discussion:** Bullae were seen in 7.2% of a healthy population up to 40 years old. Increasing age, smoking, and being male were identified as statistically significant risk factors, both in independent and in multivariate logistic regression analyses. Our observations may warrant a re-evaluation of the contribution of bullae to PBT and AGE, as the latter two occur very rarely and bullae appear to be more frequently present than earlier assumed.

## 1 Introduction

Diving, both recreational and commercial, necessitates profound physiological adaptations in humans (Bosco et al., 2018). Apart from the well-known cardiovascular effects of immersion, during descent and subsequent ascent, air-filled cavities, such as the lungs, must withstand the changes resulting from increasing and decreasing ambient pressure (British Thoracic Society Fitness to Dive Group, 2003). During ascend, pressurized air will expand, and it is of the utmost importance that the excess in volume can be exhaled. Failure to adapt to the decrease in ambient pressure can lead to severe injuries, including pulmonary barotrauma (PBT) and arterial gas embolism (AGE) (Mitchell et al., 2022). Therefore, to ensure the safety of both commercial and recreational divers, most diving organizations require (comprehensive) medical examinations (Wendling et al., 2003; Health and Safety Executive, 2023).

Most cases of PBT occur in the absence of pre-existing pulmonary pathology. However, based upon historic data the presence of structural changes associated with an air component, such as bullae/blebs or cysts, is considered to predispose to the occurrence of PBT (Tetzlaff et al., 1997). Although the exact relation between PBT and structural or functional pulmonary abnormalities is still unclear, the current guidelines advise scrutiny in evaluating the fitness to dive of patients with bullae on a case-by-case basis. Furthermore, the presence of bullae or cysts visible on chest X-ray is still considered to contraindicate scuba diving (British Thoracic Society Fitness to Dive Group, 2003).

In numerous standards, chest X-ray is still mentioned to exclude clinically relevant pulmonary pathology, but computed tomography (CT) for structural analysis of the pulmonary system has been shown a highly superior imaging technique (Health and Safety Executive, 2023). However, higher-resolution CT scans in healthy individuals can uncover incidental findings of unknown clinical significance, raising concerns for its routine use in fitness to dive assessments in the general population (Wingelaar et al., 2020; Bonnemaison et al., 2022). A forensic (*post mortem*) CT study in 130 adults without pulmonary injuries revealed that 33.8% of subjects had bullae/blebs (de Bakker et al., 2020a). Bonnemaison et al. reported a 2.3% prevalence of bullae and cysts in 307 (96% male) young military diving candidates (Bonnemaison et al., 2022). Recently, Weaver et al. reported a 4.7% prevalence pulmonary cysts, emphysema and/or bronchiectasis, based upon the analysis of the reports of almost 80,000 chest CT scans using text mining techniques (Weaver et al., 2022). Although this study provided valuable additional insights, there are limitations. In particular, the assumption that abnormalities not mentioned in the reports are truly not present.

To estimate the prevalence of bullae in the general population, we conducted a re-evaluation of a large quantity of high-quality CT scans, aiming to ascertain the presence of bullae/blebs, and to identify possible risk factors for developing bullae.

## 2 Methods

### 2.1 Design

This single-center cross-sectional observational study was conducted at OLVG, a large teaching hospital in Amsterdam, the Netherlands. The study aimed to investigate the prevalence of bullae in a (relatively) healthy population of young adults between the ages of 18 and 40, who underwent CT scans because of a clinical suspicion for pulmonary embolism (PE), during the period from 1 January 2016, to 1 March 2020. During the data collection period, two different clinical decision rules were used to determine the need for a CT scan. Up until 01-01-2018 the Wells criteria combined with a D-dimer test were used. Hereafter, the YEARS criteria combined with D-dimer were used (Hansell et al., 2008; Wang and Ji, 2020). The study end date was specifically chosen to exclude any potential additional effects of COVID-19 pneumonia, as it was not yet endemic in the Netherlands at that time.

### 2.2 Ethics and consent

Approval for the study was obtained from the ethics committee of the OLVG hospital in Amsterdam (ref number: WO 21.017). As a retrospective study, informed consent was not required under national legislation. Personal data handling and privacy adhered to the guidelines of the Association of Universities in the Netherlands and the Declaration of Helsinki.

### 2.3 Patient selection and exclusion criteria

The clinical records of selected cases were screened to ensure that no other clinical indication for the CT scans was present, and the medical history confirmed the absence of underlying pulmonary disease. Patient characteristics, including age, sex, and smoking status, were recorded for each subject. Cases were excluded if their medical history was missing, the quality of the CT scan was suboptimal (e.g., due to technical faults, extensive imaging artifacts, atelectasis, or pleural fluid obscuring more than one pulmonary lobe), or if a new pulmonary diagnosis was detected on the CT scan (e.g., previously unknown diseases such as pneumonia, emphysema, or cancer). Bullae were defined according to radiologic standards as thin-walled (<1 mm) cavities (Hansell et al., 2008). Blebs were grouped in with the bullae as a subgroup of bullae, as blebs could be classified as bullae with a direct connection to pleura. We classified all bullous air-filled anomalies as bullae either in direct contact with the pleura, or as intrapulmonary bullae. The following CT scanners were used: 2008 GE Discovery, 2015 Philips Brilliance iCT 256 slice detector, 2015 Philips Ingenuity 128 slice detector and the 2017 GE revolution scanner. The majority of scans were made using the Philips CT scanners from 2015.

### 2.4 Assessment and data collection

The included scans were assessed by the first author (MB) and scored for the presence of bullae and other abnormalities. The number, size, and location (i.e., subpleural or intrapulmonary and pulmonary lobe) were documented. All positive findings were cross-checked by a highly experienced pulmonary physician (PB). A quarter of the scans that did not show bullae were screened for possibly missed bullae by a thoracic radiologist (JW). For analyses, the collected data and baseline characteristics of the cases were recorded in a web-based database for clinical trials (Castor Electronic Data Capture, version 2022.2.0.1, Amsterdam, the Netherlands).

### 2.5 Sample size calculation and statistical analyses

A sample size calculation, based on an estimated prevalence of 5% for bullae, with a 95% level of confidence and an absolute margin of error of 2%, determined that a target population of 457 participants would provide sufficient statistical power (Wang and Ji, 2020). To ensure adequate statistical power and enhance the study’s robustness, a target of 1000 CT scans was chosen. To statistically test differences in proportions between age, sex and smoking status, Chi-square or Fisher Exact Tests were used where appropriate. For this, patients were categorized into four age groups (18–25, 26–30, 31–35, and 36–40). A multivariate logistic regression analysis was performed to study the independent predictive value of risk factors identified. Statistical analyses were performed with SPSS Statistics for Windows (IBM Corp; Armonk, NY: 2020, version 27.0). Statistical significance was assumed when alpha <0.05.

## 3 Results

**FIGURE 1:**
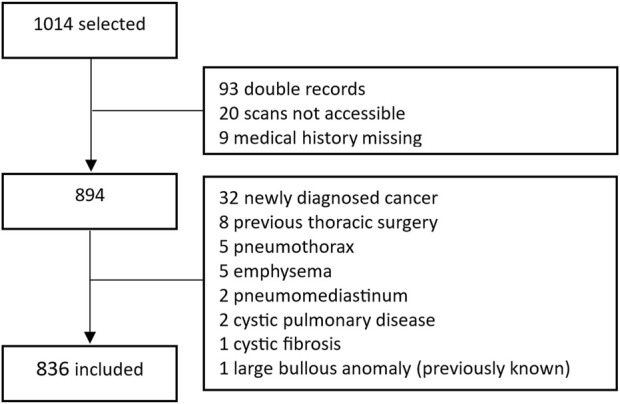
Flowchart showing inclusion and exclusion of patients in the study.

A total of 1,014 cases were identified, of which 29 were excluded because the medical history was missing, or the quality of the CT scan was insufficient for analysis. An additional 93 records were excluded because patients had multiple scans. In this case, the oldest available CT scan was used for this analysis. Another 56 were excluded because a new pulmonary diagnosis was found, therefore, 836 cases could be included in this analysis. This is visually displayed in Figure 1.

Baseline characteristics of the patients studied are shown in Table 1. Subjects were more or less equally divided over the four predefined age groups; however, in all groups, females were overrepresented and formed 74.8% of the patients studied. In 229 cases (27.3%) smoking status was not reported, but in the cases where the data were available the majority (69.5%) did not smoke.

**TABLE 1:** Baseline characteristics.

Age group	N (%)	Sex (%)	Proportion smoking^a^
18–25	183 (21.9)	Male: 52 (28.4)	0.30
		Female: 131 (71.6)	0.25
26–30	224 (26.8)	Male: 43 (19.2)	0.29
		Female: 181 (80.8)	0.25
31–35	240 (28.7)	Male: 56 (23.3)	0.43
		Female: 184 (76.7)	0.23
36–40	189 (22.6)	Male: 59 (31.2)	0.58
		Female: 130 (68.8)	0.37
Total	836 (100)	Male: 210 (25.1)	0.42
		Female: 626 (74.8)	0.27

^a^ In 229 cases the smoking status was unknown. Percentage smoking based upon known data.

### 3.1 Prevalence of bullae

**FIGURE 2:**
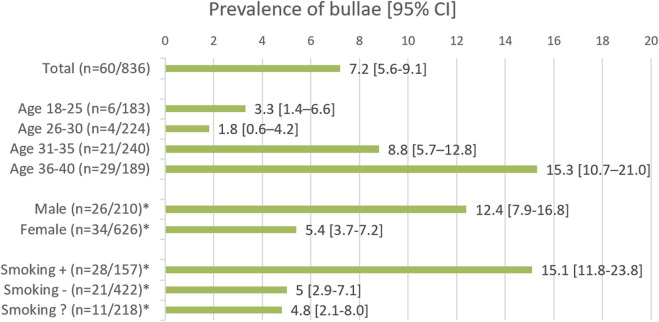
The groups marked with an asterisk (*) show the results for the age range of 18–40 years. Smoking followed by +, − and ?, represents the smoking, non-smoking, and smoking status unknown groups, respectively.

In the whole population the prevalence of bullae was 7.2% (*n* = 60/836). An overview of the prevalence in the different subgroups is shown in Figure 2. When analyzing the different age groups, a statistically significant increase in prevalence was seen with increasing age (Chi^2^ *p* < 0.001), with odds ratios (OR) of 2.829 (95% CI 1.118–7.160 *p* = 0.028) and 5.347 (95% CI 2.164–13.213 *p* < 0.001) comparing respectively the 31–35 and 36–40 groups to the youngest (18–25) group. Moreover, males had a higher prevalence than females (Chi^2^ *p* < 0.001), with an OR of 2.460 (95% CI 1.144–4.208; *p* = 0.001), as did smokers compared to non-smokers (Chi^2^ *p* < 0.001) with an OR of 3.406 (95% CI 1.878–6.157, *p* < 0.001. This is displayed in Table 2.

**TABLE 2:** Risk factors for the presence of bullae.

Age group	N cases (% of total [95% CI])	Proportion male	Proportion smoking^a^
18–25	6 (3.3 [1.4–6.6])	0.67	0.25
26–30	4 (1.8 [0.6–4.2])	0.25	0.00
31–35	21 (8.8 [5.7–12.8])	0.38	0.53
36–40	29 (15.3 [10.7–21.0])	0.45	0.72
Total	60 (7.2 [5.6–9.1])	0.43	0.57
*p*-value	<0.001	<0.001	<0.001
Odds ratio	—	2.460 (1.144–4.208)	3.406 (1.878–6.157)
		*p* = 0.001	*p* < 0.001

^a^ In 11 cases of patients with bullae the smoking status was unknown, therefore, the proportion was calculated with known data only.

In the smoking part of the population, the same pattern was seen. In the group of smokers compared to the non-smokers, a significantly higher prevalence of bullae was observed in the oldest age groups: OR 3.375 (95% CI 1.218–9.350, *p* = 0.022) in the 31–35 group, and OR 4.390 (95% CI 1.691–11.401, *p* = 0.002) in the 36–40 group. In addition, smoking males had a significantly higher prevalence of bullae than smoking females, 27.9% vs 8.9%, with an OR of 3.969 (95% CI 1.723–9.144, *p* = 0.001). In the non-smoking part of the population, a similar significant effect of sex was not found [OR 0.944 (95% CI 0.3092–2.883, *p* = 0.920)]. Likewise, a significant increase in prevalence was not seen when comparing the four different age groups to each other (Chi^2^ *p* > 0.05). However, when comparing the 18–30 group to the 31–40 group a significant increase of prevalence was seen with an OR of 2.861 (95% CI 1.088–7.524, *p* < 0.001).

To put the results into perspective, a multivariate logistic regression analysis was performed. In Table 3 the variables of the equation are displayed. The baseline of the model are females of the youngest age group, and parameters are thus compared to this baseline value. In this analysis, increasing age, the male sex and active smoking were shown to be independent risk factors for the presence of bullae.

**TABLE 3:** Odds ratios for the presence of bullae or blebs.

Parameter	Or (95% CI)	*p*-value
Baseline	—	<0.001 *
Age 26-30	0.560 (0.154–2.032)	0.378
Age 31-35	2.896 (1.133–7.400)	0.026 *
Age 36-40	4.637 (1.853–11.604)	0.001 *
Male	2.019 (1.146–3.556)	0.015 *
Smoking	2.608 (1.398–4.864)	0.003 *

Parameters are compared to the baseline, which is the age group 18–25, nonsmoking and the female sex. Statistically significant results (*p* < 0.05) are marked with an asterisk (*).

### 3.2 Number and size of bullae

**FIGURE 3:**
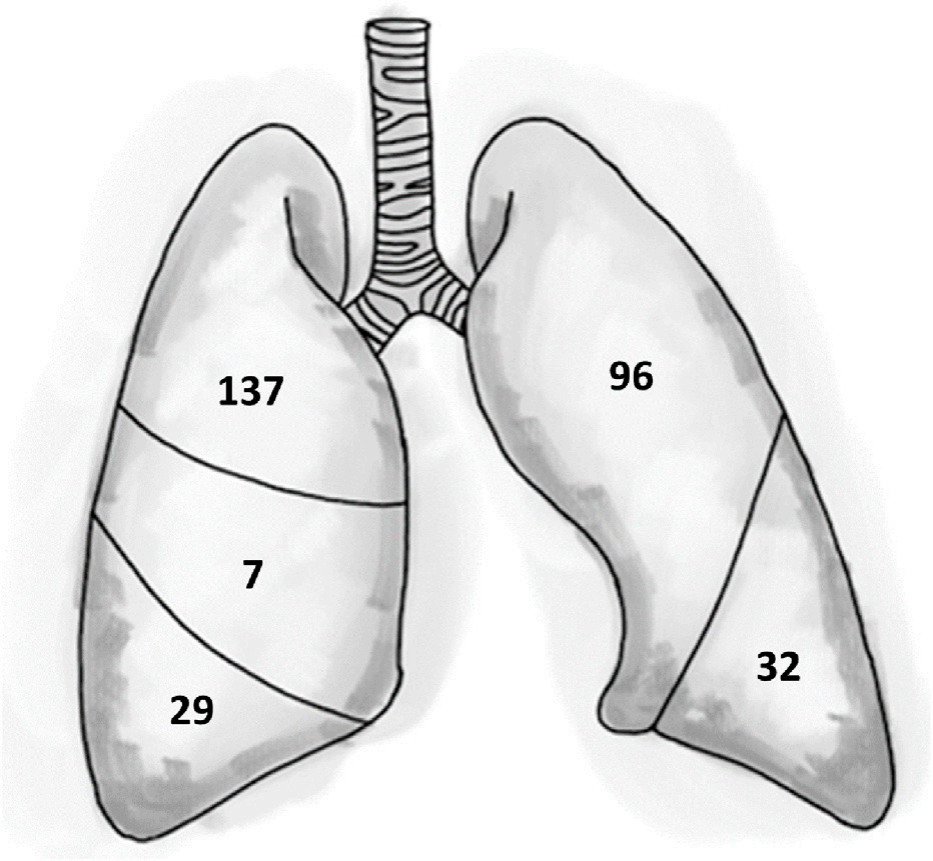
Visual presentation of location and number of bullae.

**FIGURE 4:**
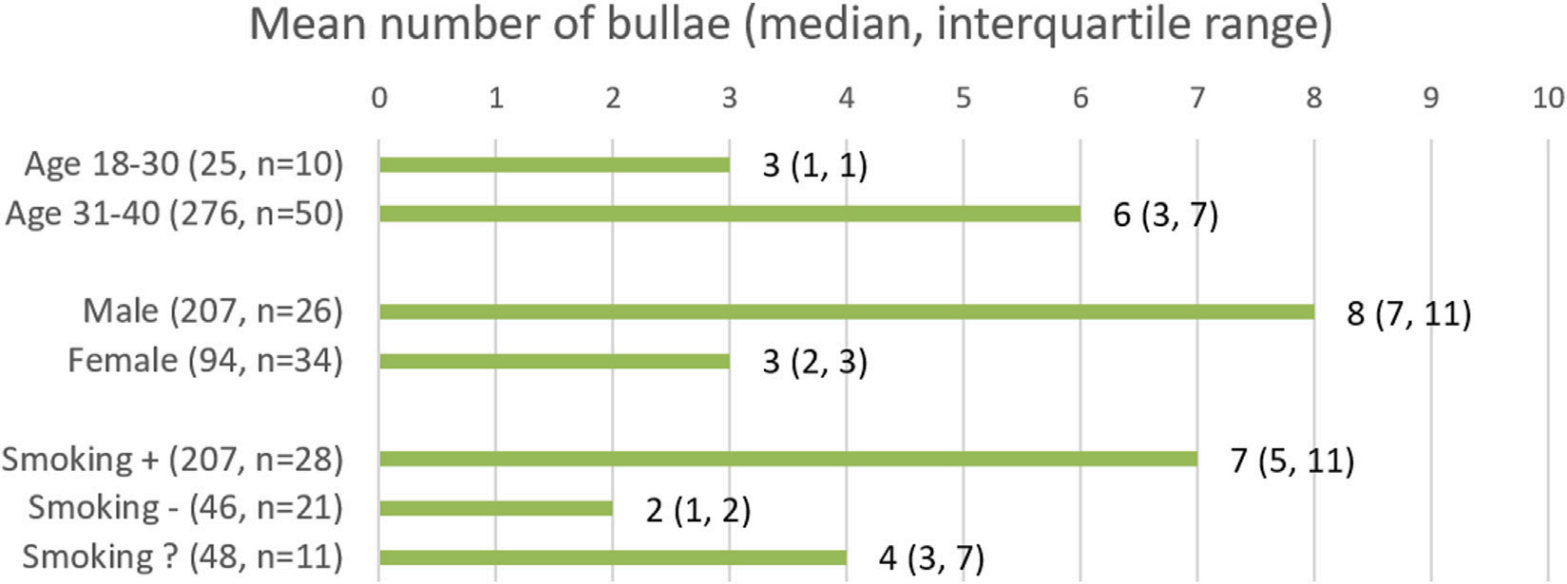
Results are rounded to the nearest integer. Smoking followed by “+”, “−“, and “?”, represents the smoking, non-smoking and smoking status unknown groups respectively*.*

Finally, we looked at the number and size of the bullae in the patients in whom bullae were identified. Bullae were present in 60/836 patients. In total, 301 bullae were found in these patients. Of these bullae, 239 were located subpleural, i.e., in direct contact with the visceral pulmonary pleura. The remaining 62 bullae were located intrapulmonary, with no relation to the pleura. The subpleural bullae had a mean size of 9.2 mm (median 6.7 mm, range 2.0–47.5 mm). Intrapulmonary bullae had a mean size of 4.9 mm (median 4.0 mm, range 2.0–17.6 mm). The majority of bullae found was found in the superior pulmonary lobes (Figure 3). As shown in Figure 4, for the different subgroups studied, in the majority of patients more than one bulla was found.

## 4 Discussion

This observational study, involving a considerable sample size, revealed that bullae are present in 7.2% (95% CI 5.6–9.1) in a healthy population under 40 years of age. The prevalence of bullae increased significantly with age, reaching 15.3% (95% CI 10.7–21.0) in the 36–40 age group, compared to 18–25 years old, with an odds ratio (OR) of 5.347. Smoking and being male were associated with higher ORs of 3.406 and 2.460, respectively. The multivariate logistic regression model also showed increased ORs, albeit slightly lower, of 2.608 for smoking and 2.109 for being male. The majority of bullae (239/301) that were identified had direct contact with the visceral pleura and had a mean size of 9.2 mm, whereas the 62 intrapulmonary bullae had mean measurement of 4.9 mm. Most of the bullae were found in the superior pulmonary lobes (233/301). In view of the high prevalence of bullae in the population studied, and the low risk of PBT in diving, over-simplification of (the interpretation of) the fitness to dive guidelines should be avoided.

Large-scale studies establishing the prevalence of bullae in the general population are lacking, and at current still impossible to conduct in the light of medical-ethical perspectives. In “general” medicine, population-level screening for bullae holds less importance since they are not usually causally linked to severe diseases and most people are not regularly submitted to great variations in ambient pressure, as experienced during diving. The role of bullae and cysts in recurrent (spontaneous) pneumothorax is well-established, but it is rarely life-threatening and can be effectively treated (Tschopp et al., 2015).

Therefore, previous investigations into the prevalence of bullae mainly originate from the field of diving medicine (de Bakker et al., 2020a; Bonnemaison et al., 2022; Weaver et al., 2022). Given their potential connection to PBT, the interest of the field of diving medicine in this topic is no surprise (Germonpré et al., 2008; Goffinet and Simpson, 2019; de Bakker et al., 2020b). However, PBT can develop without the presence of bullae due to failure to exhale during ascent (Wingelaar et al., 2018). For fitness to dive assessments, the question remains whether an individual could be assessed as fit when bullae are present, due to their unclear relation to PBT. The prevalence found in our study supports not disqualifying divers solely on the mere (radiological) presence of bullae or cysts, and raises questions about their presumed causative role in the pathogenesis of PBT. Disqualifying 15%–30% of otherwise “healthy” divers may not be justified considering the current observed low incidence of PBT, whereas breath holding during ascent may be a larger risk factor (Lafère et al., 2018). Given the rarity of PBT and AGE, in view of the higher prevalence of bullae reported, it will be essential to re-evaluate their association with dive-related injuries. In particular since there has been continuous improvement in the quality and resolution of low-dose CT imaging over the last decades, and this trend is expected to persist in the upcoming years (Withers et al., 2021). As CT images become increasingly detailed, it is logical to anticipate the discovery of more findings of unknown clinical significance in (candidate) divers in the future, which, may lead to disqualifying even more divers, using current guidelines (Wingelaar et al., 2020; Bonnemaison et al., 2022).

Consistent with previous literature, we identified being male and smoking as risk factors for the presence of bullae (Tschopp et al., 2015). Especially the male sex being identified as an increased risk factor is of interest, as most of our sample contained females. Although our study was sufficiently powered to draw conclusions, the small sample of male subjects could have distorted the “true” overall prevalence. Additionally, this study suggests that bullae may be more likely to develop with increasing age, implying the presence of yet-to-be-described risk factors; such as occupationally related risks, which are generally more present in males (Grahn et al., 2021). Alternatively, bullae might result from physiological aging or (mild) pulmonary infections. These findings also might indicate that an older CT scan does not assure fitness to dive since over time, more abnormalities might form, or pre-existing ones might increase in size. Allowing smaller “low risk” bullae to develop into larger, more dangerous bullae as long as the underlying risk factors, such as possibly smoking, are not addressed appropriately. Future studies on this important topic are warranted.

The prevalence found in our study differs slightly from the observations made by Weaver et al. This is remarkable since their prevalence also included emphysema and bronchiectasis next to bullae, as well as much older patients, with no limitations on age. Due to these factors, a higher prevalence than found in our study would be expected. As stated earlier, this difference might be because, given the fact that bullae often have no clinical relevance in normal life, bullae were not mentioned in the reports. This assumption is supported by our observation that the presence of bullae was mentioned by our radiologists in 16 out of the 60 CT scans only. Text analysis-based research on the radiologist’s rapports would have greatly underestimated the prevalence of bullae in our study. This finding could have implications for studies based on text analysis and might indicate that the true prevalence of bullae in the study of Weaver et al. might well be much higher than reported, which would be more in line with our own findings. Additionally, it could be debated whether radiologists are able to identify relevant anomalies for fitness to dive assessments without a clear statement on their potential relevance from the diving medical community.

Finally, in the majority of patients in whom bullae were detected, numerous bullae were present. Moreover, in males, smokers, and older patients more bullae per patient were present. While such data might offer insights into the development of bullae throughout life, it would not greatly alter the fitness-to-dive analysis, as now even a single bulla might be seen as a contra-indication for diving, depending on the clinician and due to the lack of a clear statement of what constitutes a high risk bulla. However, it could be the subject of future investigations to study whether the number, size and location of bullae might be a more useful predictor for PBT.

### 4.1 Strength and weakness analysis

This study represents the largest evaluation to date of CT scans with a focus on fitness to dive, providing valuable data for diving physicians when assessing recreational or commercial divers. We encourage other groups to conduct similar studies to validate or challenge our findings, further advancing the perspective on fitness to dive in the presence of bullae.

We acknowledge certain limitations in our study that warrant discussion. Firstly, the retrospective nature of the study resulted in some missing data, such as unknown smoking status in over a quarter of the cases. Additionally, coding of some subjects who had quit smoking in the past as non-smokers may have occurred. While this could potentially increase the odds ratio (OR) for smoking even further with the correct data, we consider this limitation minor. Additionally, details on the severity of smoking of the smoking population could not be adequately analysed due to insufficient data for analysis in the medical records. This information would have provided valuable further insight in the relation between more exposure to smoking and the development of bullae.

Secondly, the population used as a surrogate for the “healthy general population,” actually represented patients at the emergency department with a clinical suspicion of pulmonary embolism (PE). Although we excluded all patients with a pulmonary disease, these patients inherently differ from truly “healthy” divers without signs or symptoms (van Maanen et al., 2023). Despite this limitation, ethical considerations and radiation restrictions make it highly unlikely that a study of sufficient power in healthy volunteers will ever be conducted. Therefore, this study serves as the “best possible evidence” currently available on this topic. Additionally, the inclusion of more females than males in this study might be attributed to the clinical suspicion of PE. While this may not fully represent the predominantly male diving population, the male sample size remains statistically sufficient for drawing valid conclusion.

Lastly, the initial analysis of the CT scans was performed by one assessing researcher. One could argue that this should have been done by at least two researchers on the whole dataset to improve accuracy. However, all positive findings were confirmed by a second experienced researcher, and a quarter of the negative scans were checked by a thoracic radiologist for missed findings. This is in line with recommendations for checking data integrity with a low risk of faulty or corrupt data. With only two scans out of the checked 194 scans showing bullae that were previously coded as no bullae, this assumption proved to be correct. Indicating that by checking all data the true prevalence of bullae in our sample might be even slightly higher than reported, which further substantiates the findings in this paper.

## 5 Conclusion

In conclusion, we observed a 7.2% (95% CI 5.6–9.1) prevalence of bullae in a healthy population up to 40 years old. Increasing age, smoking, and being male are identified as statistically significant risk factors, with ORs of 5.347 (36–40 compared to 18–20), 3.406 and 2.460. In the multivariate logistic regression model, the ORs remained significant, with values of 2.608 for smoking and 2.019 for being male. Modern guidelines for fitness to dive, while urging for caution, do not systematically exclude divers with bullae from diving, but evaluate case by case. The high prevalence of bullae, as revealed by this study, warrants further research to clarify their relevance and contribution to the development of PBT, especially given that the diving population is typically older and still predominantly male compared to our study cohort. We encourage other research groups to replicate similar studies to either validate or challenge our findings. Moreover, future research endeavours should further study the potential predictive value of additional parameters, such as the number, location, size of bullae, and relation with packyears, to gain deeper insights into which bullae may be more prone to contribute to the development of PBT.

## Data Availability

The datasets presented in this article will be made available upon reasonable request. Requests to access the datasets should be directed to the corresponding author.
